# Pseudocapacitive materials for energy storage: properties, mechanisms, and applications in supercapacitors and batteries

**DOI:** 10.3389/fchem.2025.1636683

**Published:** 2025-06-27

**Authors:** Yi-Min Wei, Kulurumotlakatla Dasha Kumar, Long Zhang, Jian-Feng Li

**Affiliations:** ^1^ 21C Innovation Laboratory, Contemporary Amperex Technology Co. Limited, Ningde, China; ^2^ College of Chemistry and Chemical Engineering, State Key Laboratory of Physical Chemistry of Solid Surfaces, The MOE Key Laboratory of Spectrochemical Analysis and Instrumentation, College of Energy, College of Materials, Xiamen University, Xiamen, China; ^3^ Innovation Laboratory for Sciences and Technologies of Energy Materials of Fujian Province (IKKEM), Xiamen, China

**Keywords:** pseudocapacitor, electrode materials, metal oxides, electrochemical properties, charge storage mechanisms

## Abstract

The growing demand for efficient energy storage has intensified interest in pseudocapacitive materials, known for their high-power density, rapid charge–discharge capabilities, and tunable physicochemical properties. This review explores the foundational principles and evolution of pseudocapacitive materials, emphasizing recent strategies to improve their electrochemical performance in supercapacitor applications. Key focus areas include: 1) intercalation-type materials such as Nb_2_O_5_, TiO_2_, and V_2_O_5_, which offer fast and reversible ion insertion without phase transitions; 2) redox-active materials like transition metal oxides and 2D materials (e.g., MXenes), which enhance charge storage through surface and near-surface Faradaic reactions; and 3) materials relying on surface adsorption mechanisms that enable ultrafast kinetics and excellent cycling stability. Special attention is given to nickel-based compounds NiO, Ni(OH)_2_, and related composites owing to their high theoretical capacitance, multiple valence states, and cost-effectiveness, making them promising for both supercapacitors and hybrid energy storage devices. The interplay between structural design, conductivity, and electrochemical behavior is critically assessed. Lastly, the review outlines current challenges and future directions in the development of scalable, high-performance pseudocapacitive materials. This work aims to guide the rational design of next-generation electrode materials for advanced supercapacitor technologies.

## Graphical Abstract

**Figure:**
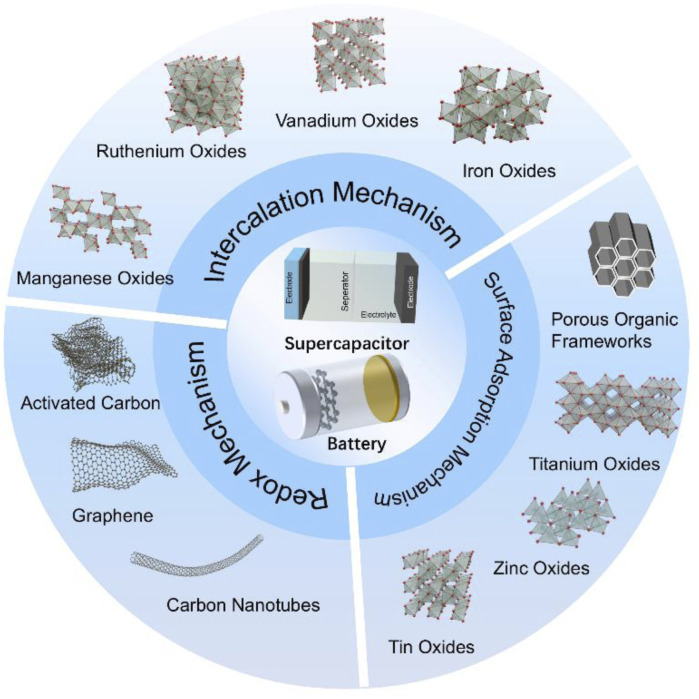
This review summarizes the basic principle and fundamentals of pseudocapacitive materials, highlighting recent examples of their applications and their superior electrochemical properties in supercapacitors and batteries.

## 1 Introduction

**FIGURE 1:**
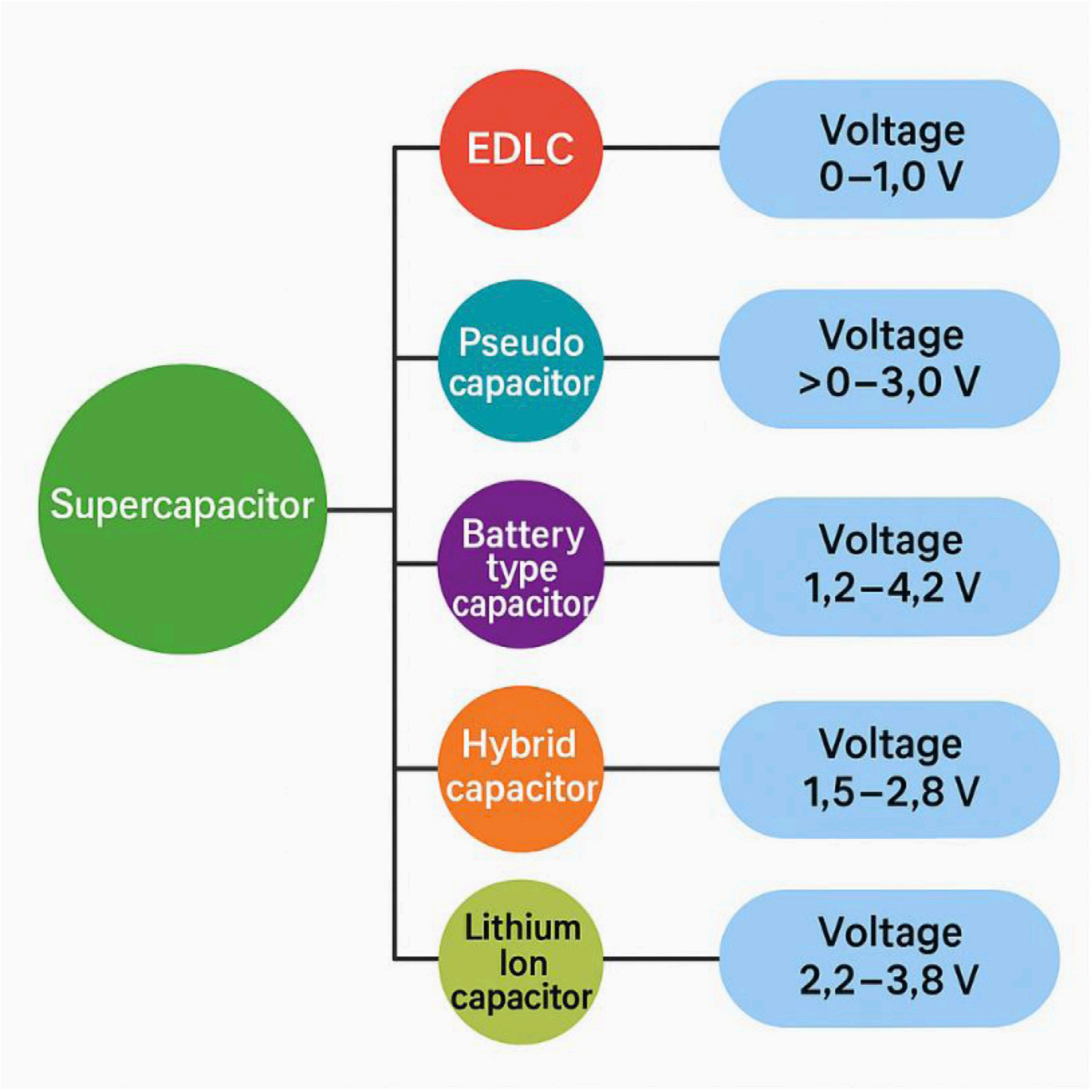
Shows a varieties Supercapacitors battery: hybrid, battery-type, electric double-layer capacitors (EDLCs), and pseudo capacitors. Reprinted with permission from Bhat et al. (2023).

Worldwide energy requirements and conservation efforts have grown since the industrial age, highlighting a pressing need to address social and environmental issues over the last 5 decades (Yang et al., 2011; Chu and Majumdar, 2012). As a result, the reliance on fossil fuels has declined since the 1970s, whereas the demand for renewable and sustainable energy sources has surged (Jiang et al., 2020; Gür, 2018). For renewable energy sources like solar, wind, and geothermal to stabilize their intermittent nature, energy-storage systems (ESSs) are essential. When compared to fossil fuels, electrochemical conversion and storage of renewable energy have a significant effect (Noori A. et al., 2019; Blanco and Faaij, 2018). Supercapacitors (SCs) have exceptional energy storage capabilities, including low cost, long cycle life, high power density, specific capacity, rapid charge/discharge time, and operational stability (Castro-Gutiérrez et al., 2025; Huang et al., 2017). SCs are classified into two types according to their energy storage methods: electrochemical double-layer capacitors (EDLCs) and pseudocapacitors. Currently, pseudocapacitors are gained greater attention since, in comparison to EDLCs, the energy density related to the faradaic process is significantly larger. Therefore, significant efforts are made to create novel designs and prepare electrode materials based on pseudocapacitors with the goal of improving the electrochemical characteristics for energy storage. Pseudocapacitors are increasingly being used as a remedy for these issues (Zhang et al., 2018; Simon and Gogotsi, 2008). Recently designed electrochemical capacitors have demonstrated superior energy density in comparison to conventional carbon-based electrical double-layer capacitors (EDLCs) (Kavaliauskas et al., 2011). The advantages are larger voltage window and increased capacity of the Faradaic electrode. On the other hand, pseudocapacitors to perform faster reversible redox reactions, they have a higher energy density, unlocking the door to potential large-scale uses. Transition metal-based hydroxides and oxides have been thoroughly studied as potential electrode materials for high-performance pseudocapacitors (Wang et al., 2015; Peng and Zhang, 2023). For instance, research has been done on electrodes like NiO (Burke, 2000), RuO_2_ (Anderson et al., 2010), Co_3_O_4_ (Frackowiak and Beguin, 2001), MnO_2_ (Wang et al., 2012), Ni(OH)_2_ (Arbizzani et al., 1996), and Co(OH)_2_ (Mala et al., 2023), as well as their compounds. The Rich redox reaction sites associated with bimetallic nickel-based hydroxides have recently been demonstrated to have substantial theoretical specific capacitance values, indicating that these materials have great potential as batteries (Jayakumar et al., 2024). Despite their rapid charging and discharging times, supercapacitors have lower energy densities (5 Wh kg^−1^) than batteries; nevertheless, they can achieve a far higher power density (10 kW kg^−1^) in the same amount of time (Liu et al., 2023; Yan et al., 2023). SCs are therefore viewed as a viable alternative to batteries, especially in the area of energy appliance load lifting and various types of storage batteries electrochemical performances as shown in Figure 1. However, meeting the growing demand for future arrangements necessitates substantial advancements in supercapacitor performance as new materials are developed and the scope of nanoscale electrochemical alliances is expanded (Shoeb et al., 2023; Hou et al., 2023).

Over the last 10 years, major improvements have been achieved an experimental and theoretical study of SCs, including the development of various SCs material categories, device fabrication types, and electrolytic compounds. The production of next-generation energy storage materials requires a special method for creating novel materials. Developing novel materials with unique morphologies and stable crystal structures essential for SCs, batteries, hydrogen storage, and other applications aim of contemporary synthetic chemistry. Therefore, electrode materials with suitable properties are needed for high-performance ESS systems. Reviews on these subjects are quite good (Hu and Wang, 2002). Among the most promising candidates for advanced SCs are transition metal oxides and 2D materials, such as MXenes, metal–organic frameworks (MOFs), and covalent organic frameworks (COFs). These emerging materials exhibit exceptional potential for a wide range of energy storage applications (Li and Zhitomirsky, 2013). Pseudocapacitive materials derived from these systems offer enhanced energy storage capabilities due to their multiple accessible oxidation states. Additionally, their tunable porosity and hierarchical architectures can be strategically engineered to improve ion transport and achieve high energy densities. However, several challenges hinder their widespread application in supercapacitor systems, including limited electrical conductivity, structural instability during cycling, and complex synthesis routes. Overcoming these limitations is critical for the development of high-performance, durable, and scalable energy storage devices.

This review focusses on current research into pseudocapacitive electrode materials for SC applications. We begin by discussing the origins of pseudocapacitance and the fundamental principles of SCs, followed by characteristics of pseudocapacitive materials and pseudocapacitive electrodes. Next, we examine the mechanism of intercalation, redox and surface adsorption of pseudocapacitance electrode materials. Particularly, we discuss the following categories: 1) intercalation-type materials such as Nb_2_O_5_, TiO_2_, and V_2_O_5_, highlighting their structural features and electrochemical performance; 2) redox-active materials, including metal oxides (MnO_2_, Fe_3_O_4_, and V_2_O_5_) as well as MXenes and metal-organic frameworks (MOFs), which play vital roles in enhancing charge storage via reversible redox reactions; and 3) surface adsorption mechanism-based materials, involving functionalized 2D materials and porous organic frameworks (POFs), contribute to high-rate capability and excellent cycling stability through hydrogen bonding at the electrode–electrolyte interface. Furthermore, Ni-based materials are highlighted as highly promising electrode material for SCs. They offer several unique characteristics, like cost-effective cost, friendly to the environment, the availability in nature, and high theoretical capacitance. Ni-based electrodes also exhibit high specific capacitance, reversible redox activity, and good electrochemical stability, making them suitable for high-performance energy storage systems. This review aims to provide insights into strategies for achieving optimal material properties for pseudocapacitive applications. We hope it will guide future research toward leveraging the intrinsic characteristics of advanced materials and identifying the most effective systems for next-generation energy storage technologies.

## 2 Understanding pseudocapacitors: principles and progress

The first carbon-based (NEC) Supercapacitor (SC) electrode, known as “Supercap,” entered the market after 10 years of arduous work by various businesses, including NEC and Sohio, to create commercial products beginning in the 1970s. Research on the alleged “pseudocapacitors” started around 1980. These devices are entirely new and operate in a different manner (Wu et al., 2007; Zhang et al., 2009). The simultaneous “double layer” and faradaic reactions, which combine the functions of electrochemical batteries and capacitors, allow for electrical charge storage. Thin layers of transitional metal oxide active materials were deposited on metallic substrates to create these devices.

More recently, enhanced activated carbon-based SCs were used to create high-voltage electrolytes (such as organic and ionic liquids) (Garche, 2024), which allowed the market to meet demands for high-power devices. It is important to note that, since then, the number of categories and definitions has increased steadily, resulting in a state of near-anarchy in the field of SCs (Ganesh et al., 2006). The electrolyte is impregnated into the two electrodes, which are separated by a membrane separator that promotes ion mobility and inhibits electric contact. These devices have a high degree of recyclability due to the usual electrostatic charge transfers (Joseph and Thomas, 2023). New porous and conductive electrode materials have high specific surface areas, which contribute to the increased capacitances of SCs.

In fact, the EDLC is only responsible for a small percentage of the charge; faradaic mechanisms (electrosorption, redox reactions, and intercalation) are used to transport and store a much greater quantity of charge. pseudocapacitors store an electrical charge (and thus energy) using a different process. Their electrodes are made of redox active materials. A pseudocapacitor’s electrode undergoes quick and reversible redox reactions involving the transfer of charges between the electrode and electrolyte when an external voltage is applied. A pseudocapacitor’s charge and discharge mechanism is comparable to that of batteries (Mishra et al., 2022; Wu et al., 2024). The reaction at the cathode during the charging phase could be explained by Equations 1, 2 as follows:

$$E1+A^{‐}\to \text{ charge }E1^{\delta +}\;//\;A^{‐}+\delta e^{‐}$$ (1)

Moreover, the reaction at the anode is represented by:

$$E2+C^{+}+\delta e^{‐}\to \text{ charge }E2\delta^{‐}//\;C^{+}$$ (2)

The cathode and anode are denoted by E1 and E2, the anion and cation by A^−^ and C^+^, respectively, and the electrolyte–electrode interface by//. The electrosorption valence, which is associated with oxidation-reduction reactions, is represented by the value δe^−^.

**FIGURE 2:**
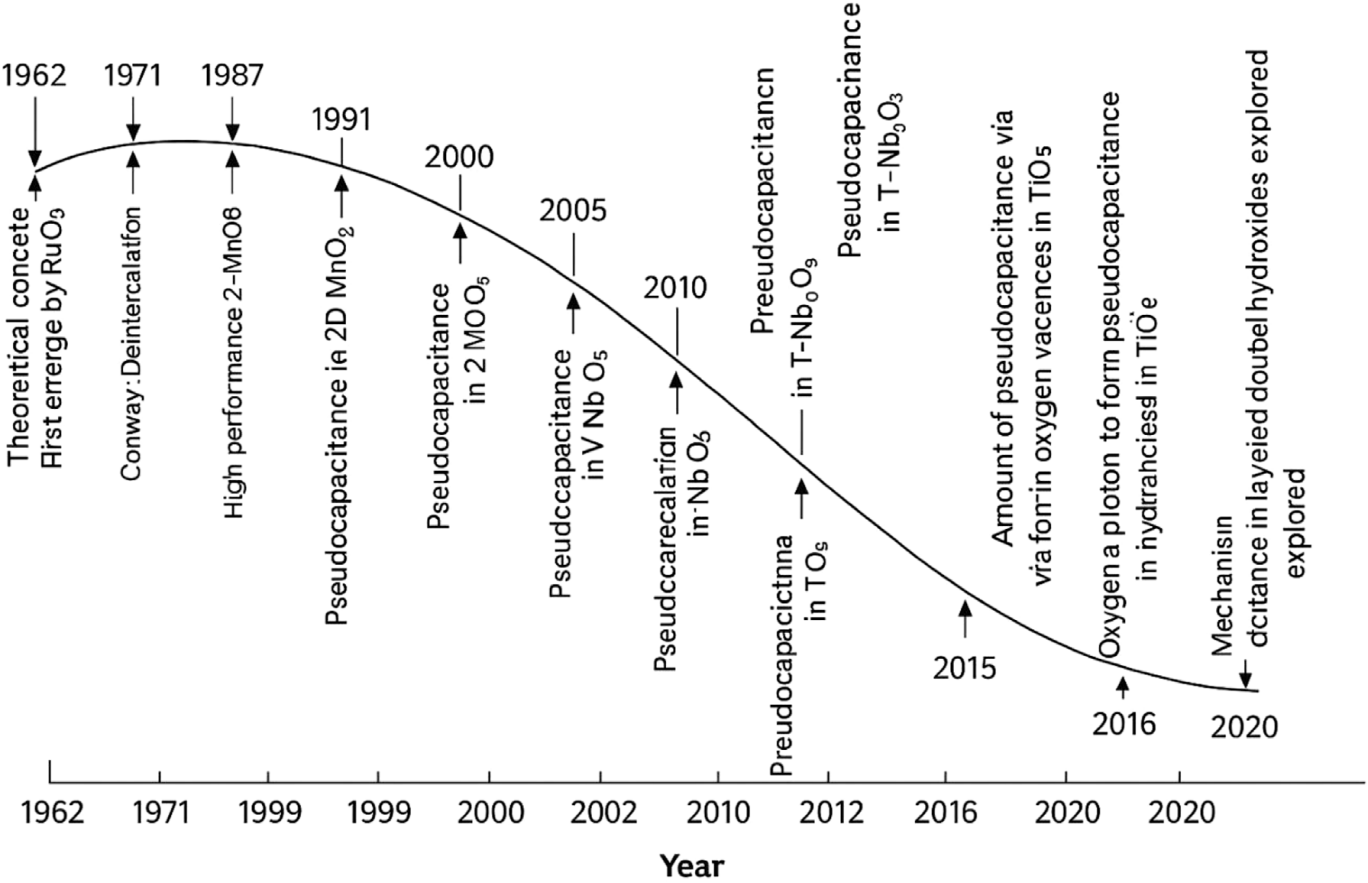
Illustrate the overall each year development of materials, mechanism, and theoretical aspects of the pseudocapacitance. Reprinted with permission from Bhojane (2022).

Pseudocapacitors offer significantly higher energy density nearly twice that of EDLCs, due to their Faradaic charge storage mechanism, which utilizes both the surface and the bulk of electrode materials. Initially, ceramic compounds like IrO_2_, RuO_2_, and NiO were explored, but in recent years, attention has expanded to include conducting polymers and alternative metal oxides (Conway and Gileadi, 1962; Conway and Angerstein-Kozlowska, 1981), aiming to enhance performance while reducing production costs. Although materials such as conducting polymers and metal oxides deliver high Faradaic capacitance, they often suffer from limited stability over long-term cycling. (Bhojane, 2022). Identified three key Faradaic mechanisms responsible for improved capacitance: underpotential deposition, redox pseudocapacitance (e.g., in RuO_2_·nH_2_O), and intercalation-based pseudocapacitance. Figure 2 shows an overall growth and understanding of mechanism, and theoretical aspects of pseudocapacitance for SCs applications.

Advantages of supercapacitors must be acknowledged in order to mitigate the drawbacks and improve mechanical strength and energy in supercapacitors applications. In these applications where integrated structures are not regularly replaced, supercapacitors are advantageous due to their long longevity and noticeably greater cycle life than batteries. First, the limitation for supercapacitor materials is compromising between specific capacitance and mechanical strength. The electrodes were treated with chemical activation processes at high temperatures to increase the electric double layer capacitance, but the treatment decreased structural strength because of damaged electrodes in nickel-based batteries. Second, a supercapacitor (SC) is one of the better energy storage alternatives that the research community is currently investigating due to the drawbacks of certain battery types materials. SCs provide energy rapidly and effectively, making them suitable for a wide range of applications. These are suitable for large-scale generate and used for low-cost and simple design in energy storage applications.

Pseudocapacitive materials are notable for their potential in designing innovative electrode systems for energy storage technologies. The field of materials chemistry is central to enhancing charge storage performance by developing such advanced materials. Electrochemical energy storage (EES) involves the conversion of chemical energy into electrical energy through redox reactions. In these systems, energy is stored within the chemical bonds of electrode materials used in batteries and pseudocapacitors (Trasatti and Buzzanca, 1971) Effective energy storage methods depend on electrodes that offer excellent electrical and ionic conductivity, along with high power and energy densities. The following problems will be addressed by the material’s chemistry design for the EES: 1) Intercalation materials such as Nb_2_O_5_, TiO_2_, and V_2_O_5_ enhance electrochemical performance by enabling fast and reversible ion insertion without compromising structural stability. 2) Redox mechanisms in 2D materials include Mxenes and metal-organic frameworks (MOFs), which offer high conductivity and rich surface chemistry for efficient charge storage. 3) Surface adsorption in pseudocapacitors involves functionalized 2D materials that provide abundant active sites for enhanced ion interaction and charge storage. Thus, the structure-property materials are helping to improve properties and electrochemical performance. When it comes to element selection, metal cations that can transmit multiple electrons per metal cation can be chosen to create high energy density electrode materials. The structure’s design and selection should provide high stability and high-rate capability; to accomplish this, the crystal structure must be compact and strong, and the chemical bonding must be strong enough to withstand the severe and demanding testing that occurs during electrochemical reactions (Gogotsi and Penner, 2018).

The faradic law, which follows surface redox and intercalation, is caused by the charge transformation reaction involved in the energy storage mechanism. Surface absorption, surface redox, and intercalation all involve the thermodynamic and kinetic behaviour of electrosorption/desorption, resulting in pseudocapacitance (Costentin et al., 2017; Dubal et al., 2015). Electrode surface interactions with reactive species typically occur in two modes: one involves repulsive interactions, leading to peak broadening in cyclic voltammetry, often associated with Frumkin-type electrosorption (characterized by a broad peak); the other entails attractive interactions that result in redox activity and follow Langmuir-type electrosorption, evident as sharp peaks in the voltammetric curve (Devaraj and Munichandraiah, 2008). Ruthenium dioxide (RuO_2_) was the first compound recognized for exhibiting pseudocapacitive charge storage behavior (Liu and Anderson, 1996). In neutral electrolytes, hydrated manganese dioxide (MnO_2_·H_2_O) demonstrated capacitive characteristics (Kamila et al., 2019). Unlike electric double-layer capacitors (EDLCs), pseudocapacitors utilize redox reactions that allow access to multiple oxidation states, thereby achieving higher energy densities (Gao et al., 2010; Zhou J. Ye et al., 2020). Asymmetric supercapacitor cells can enhance energy density more effectively when the battery-type electrode facilitates faster electron transport, while the capacitive electrode stores charge electrostatically. This synergy leads to improved charge transfer kinetics, particularly under high current conditions (Wang et al., 2005). Extensive research is ongoing into transition metal oxide-based materials, such as nickel oxide (NiO), vanadium pentoxide (V_2_O_5_), cobalt oxide in spinel form (Co_3_O_4_), iron oxide (Fe_2_O_3_), and binary spinel compounds like NiCo_2_O_4_ for their potential in pseudocapacitor applications (Samantara et al., 2018; Huai et al., 2023). However, transition metal oxide-related structural instability and performance degradation concerns prompt research into innovative framework structures for improved structural stability and increased surface charge storage (Mishra et al., 2021; Zhang et al., 2015).

Metal-organic frameworks (MOFs) are formed by coordinating metal ions with organic ligands, resulting in fascinating two- or three-dimensional networks that exhibit permanent porosity (Saraf et al., 2016). These porous frameworks often display faradaic pseudocapacitive behavior, particularly when oxalate linkers are involved, as they can actively engage in redox reactions with metal centers (Han et al., 2018; Yang et al., 2014). However, many transition metal oxalate-based structures tend to retain water molecules within their framework due to their open architecture, which facilitates water absorption. To address this, we propose a strategy involving the controlled elimination of these structural water molecules. This approach aims to produce a tailored porous network capable of supporting enhanced charge storage by enabling efficient anion intercalation and deintercalation, alongside electric double-layer capacitance. Maintaining the intrinsic porosity during water removal is crucial, as it supports rapid ion diffusion and charge transport, ultimately leading to superior capacitive performance. Pseudocapacitors, a subclass of electrochemical capacitors, differ significantly from (EDLC) in their charge storage mechanism. In contrast to electric double-layer capacitors (EDLCs), which store energy via electrostatic charge buildup at the interface between the electrode and electrolyte, pseudocapacitors utilize rapid and reversible Faradaic redox reactions near or on the electrode surface for energy storage. These reactions typically involve electron exchange between the electrode and electrolyte, often coupled with ion adsorption or insertion into the electrode material. As a result, pseudocapacitors can deliver higher specific capacitance and energy density compared to EDLCs, while retaining relatively fast charge–discharge capabilities. The overall charge storage behavior in pseudocapacitors can be categorized into three main mechanisms: redox pseudocapacitance, intercalation pseudocapacitance, and surface adsorption (electrosorption).

## 3 Overall mechanisms of pseudocapacitance in electrode materials

In supercapacitors, pseudocapacitance performs excellent electrochemical charge storage mechanism in various metal oxides, 2D materials like Mxene, metal–organic frameworks (MOFs) and covalent organic frameworks (COFs) to overcomes the electrostatic ion accumulation seen in EDLCs. Unlike electric EDLCs, which rely solely on electrostatic ion accumulation, pseudocapacitors overcome this limitation by enabling faradaic processes.

**FIGURE 3:**
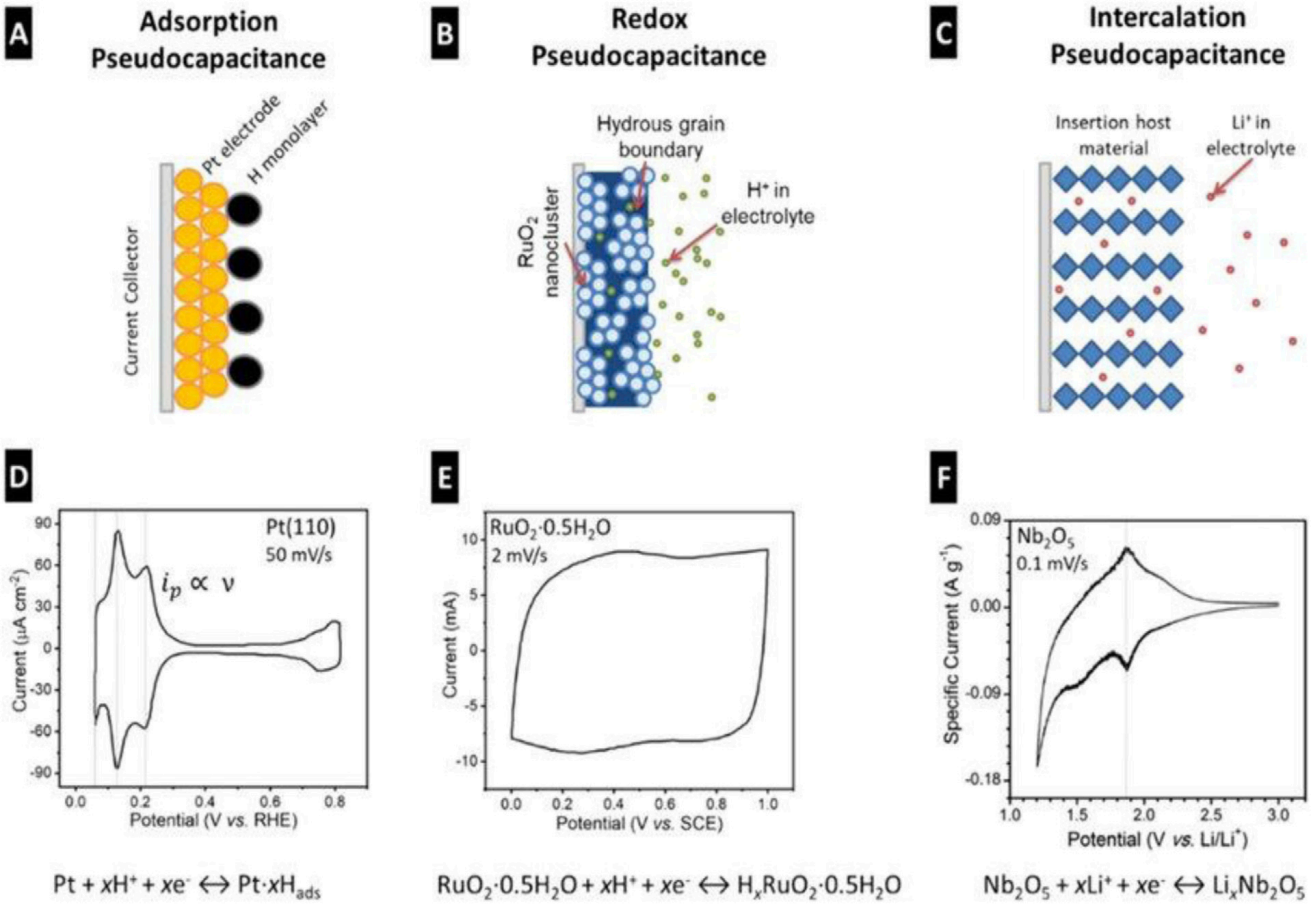
Charge storage mechanisms and cyclic voltammograms of different pseudocapacitor types as classified by Conway: **(A–D)** surface adsorption, **(B–E)** redox reactions, and **(C–F)** ion intercalation. Reprinted with permission from Hu et al. (2015).

Pseudocapacitive behavior involves intercalation, redox reactions, and electrosorption, with rapid and reversible redox processes occurring at or near the electrode surface. The development of emerging pseudocapacitive materials can be broadly classified into three categories based on their charge storage mechanisms (as shown in Figure 3): 1) Intercalation-based pseudocapacitance, which combines the benefits of both capacitive and battery-like behaviour, enabling high-performance energy storage; 2) Redox-actice pseudocapacitance, wherein materials facilitate rapid and reversible redox reactions at or near their surfaces, contributing to efficient charge storage; 3) Electrosorption-driven pseudocapacitance, which has recently seen advancements through the use of functionalized carbon materials that enhance ion adsorption and surface interaction.

### 3.1 Mechanism of intercalation pseudocapacitance and electrode materials

The majority of these materials are energy storage materials based on intercalation. The materials that have been studied the most and laid the foundation for a basic understanding of pseudocapacitance are typical pseudocapacitive materials such as hydrous RuO_2_·nH_2_O, IrO_x_, ZnO, Nb_2_O_5_, TiO_2_, etc. (Wang et al., 2018; Duddi et al., 2025). Traditional pseudocapacitive electrode materials offer a tempting compromise between EDLCs and batteries, but they also have their own set of disadvantages. Metal hydroxides and oxides are not good electrical conductors by nature. These materials may undergo notable volume changes as a result of the frequent insertion and extraction of ions during charge/discharge cycles (Ramachandran et al., 2024). Balancing acceptable electrochemical performance while achieving large mass loading of the active material on the current collector is challenging. Increased ion diffusion distances and decreased power capabilities may affect the electrodes. Reversible ion doping and dedoping are essential for conducting polymers’ conductivity and electrochemical activity (Fan et al., 2018). The functional groups that generate pseudocapacitance may diminish or separate with prolonged cycling, which could lower the pseudocapacitive contribution and overall performance. Aiming to create composite materials, enhance nanostructures, create novel materials, and study different electrolytes, current research in the field is concentrated on these boundaries. Importantly, while both Li-ion batteries and pseudocapacitive materials may utilize intercalation as a charge storage mechanism, their fundamental behaviors are distinct. In conventional Li-ion batteries, intercalation typically involves diffusion-limited processes and often results in phase transitions within the electrode materials. These reactions, while providing high energy density, are generally slower and may lead to structural strain, limiting their rate capability and cycling stability. On the other hand, pseudocapacitive intercalation also referred to as intercalation pseudocapacitance is characterized by highly reversible ion insertion into the electrode’s bulk without undergoing classical solid-state phase transformations. This results in charge storage that is surface-like in kinetics but bulk-like in thermodynamics.

Intercalation pseudocapacitance is a hybrid charge storage mechanism where ions insert into the bulk of an electrode material in a highly reversible manner and at a rate comparable to surface redox reactions. This is made possible by the availability of open crystal frameworks, short diffusion lengths, and facile ion migration channels that allow ions (e.g., Li^+^, Na^+^, H^+^) to be rapidly intercalated into specific crystallographic sites (Aghazadeh and Foratirad, 2022; Salanne et al., 2016; Li et al., 2017). The charge storage in intercalation pseudocapacitance is thus both capacitive in kinetics and battery-like in thermodynamics, enabling high specific capacitance and fast response. One of the most well-studied materials exhibiting intercalation pseudocapacitance is orthorhombic Nb_2_O_5_. Augustyn et al. showed that this material stores charge by ultrafast lithium intercalation into its open channels without undergoing a phase change, allowing for rapid kinetics and high power density. Their study reported a specific capacitance of 200 F/g^−1^ and outstanding rate capabilities, with up to 80% capacitance retention at 10 A/g in Li^+^ electrolytes (Augustyn et al., 2013). This mechanism is governed by the equation: Nb_2_O_5_ + xLi^+^ + xe^-^ ↔ Li_x_Nb_2_O_5_, This highly reversible intercalation process exemplifies the non-diffusion-limited behavior characteristic of pseudocapacitive intercalation. The distinguishing feature of this system is the absence of phase transitions and minimal lattice expansion during cycling, which enhances both kinetics and stability. Mxenes such as Ti_3_C_2_T_x_ also demonstrate intercalation pseudocapacitance, particularly in hydrated or delaminated forms. In acidic aqueous electrolytes, cations like H^+^ or Li^+^ can intercalate between the layers of Ti_3_C_2_T_x_ without disrupting its layered structure. This behavior is attributed to a combination of electric double-layer capacitance and intercalation pseudocapacitance, with total volumetric capacitance exceeding 1,500 F/cm^3^. The surface terminations (–OH, –O, –F) on Mxenes actively participate in charge storage and facilitate cation transport. The pseudocapacitive intercalation is described by: Ti_3_C_2_T_x_ + xH^+^ + xe^-^ ↔ Ti_3_C_2_T_x_ (H)_x_, where the inserted protons are accommodated without crystallographic restructuring. The layered structure, coupled with high electrical conductivity and chemical tunability, makes Mxenes one of the most promising platforms for intercalation-based supercapacitors. In addition to Nb_2_O_5_ and Mxenes, materials like amorphous or nanosized TiO_2_ and V_2_O_5_ have also been identified as intercalation pseudocapacitive materials (Breitsprecher et al., 2018). Amorphous TiO_2_ allows for fast Li^+^ intercalation due to its disordered structure, which reduces ion migration barriers. V_2_O_5_, on the other hand, can accommodate multiple cations in its layered framework, enabling multivalent ion intercalation. Kim et al. demonstrated that V_2_O_5_ aerogels exhibited high-rate pseudocapacitive behavior in Na^+^-based systems, achieving a specific capacity of 300 mAh/g with excellent rate capability. Such behavior is particularly promising for emerging Na-ion and Zn-ion hybrid capacitors, where ionic size and solvation effects limit performance in traditional systems.

Overall, intercalation pseudocapacitance bridges the gap between surface-controlled pseudocapacitance and bulk intercalation seen in batteries. The design of materials with short diffusion paths, open frameworks, and structural resilience is key to maximizing performance (Qui et al., 2016; Gao et al., 2020; Fleischmann et al., 2020). Transition metal oxides (e.g., Nb_2_O_5_, TiO_2_, V_2_O_5_), 2D materials (Mxenes), and hybrid systems are continually being engineered to harness this mechanism. By enabling high energy and power densities alongside stable cycling, intercalation pseudocapacitance is emerging as a cornerstone of next-generation electrochemical energy storage.

### 3.2 Redox pseudocapacitance: mechanism and insights

Redox pseudocapacitance arises from fast and reversible Faradaic redox reactions at or near the surface of electroactive materials. Unlike electric double-layer capacitors (EDLCs), which store charge through electrostatic accumulation at the electrode/electrolyte interface, redox pseudocapacitors depend on electron transfer processes involving electrochemical oxidation and reduction. This allows for higher specific capacitance and energy density, as charge is stored both through surface redox processes and intercalation mechanisms. Pseudocapacitive redox behavior is defined by a linear charge-potential relationship across a wide voltage range, offering fast kinetics and good cyclability. This mechanism primarily involves redox-active materials like transition metal oxides, where surface reactions allow for the insertion of protons or other cations into the material’s lattice, crucially without significant phase transitions. For instance, the well-documented redox reaction for RuO_2_ in acidic media is: RuO_2_+xH++xe−↔RuO_2_−x(OH)x. This reaction exemplifies proton-coupled electron transfer, enabling high capacitance due to multiple accessible oxidation states. As reported by (Conway and Gileadi, 1962), RuO_2_ exhibits almost ideal pseudocapacitive behavior with fast charge propagation, making it a benchmark for other redox-active materials. However, owing to its extreme cost and toxicity, there is a strong research impetus to explore alternative materials with comparable electrochemical performance (Hu et al., 2015; Shen et al., 2018; Kuntoji et al., 2024). Recent research highlights metal oxides like MnO_2_, Fe_3_O_4_, and V_2_O_5_ as crucial for pseudocapacitor design. Among these, MnO_2_ is extensively investigated due to its abundance, environmental friendliness, and varied oxidation states (Mn^4+^/Mn^3+^/Mn^2+^) (Xu et al., 2017). As reported that nanostructured MnO_2_ integrated with graphene yielded a high specific capacitance of ∼1100 F/g^−1^, attributed to enhanced electrical conductivity and increased accessible surface area. Similarly, Fe_3_O_4_-based composites have been shown to exhibit energy densities above 30 Wh/kg while maintaining good capacity retention over thousands of cycles (Niu et al., 2019).

Beyond these emerging classes, 2D materials have highlighted the promise of Mxenes, particularly Ti_3_C_2_T_x_, as next-generation redox-active materials. These 2D transition metal carbides and nitrides exhibit metallic conductivity, tunable surface chemistry, and abundant terminal groups (–OH, –F, –O), which participate in redox processes. Demonstrated that Ti_3_C_2_T_x_ Mxene could achieve volumetric capacitance values as high as 1,500 F/cm^3^ in acidic electrolytes, facilitated by reversible surface redox reactions involving terminal groups and interlayer cation intercalation. Moreover, the hydrophilic and layered morphology supports fast ion diffusion, enhancing rate capability and cycling life. Covalent Organic Frameworks (COFs) represent another emerging class of pseudocapacitive materials (Sheberal et al., 2017a). Their crystalline, porous structures with tunable functional groups allow incorporation of redox-active moieties such as anthraquinone or pyrene (Jiang et al., 2020). (Chen et al., 2020) synthesized a redox-active COF (DAAQ-TFP) that exhibited stable electrochemical performance with a specific capacitance of 350 F g^−1^, owing to the reversible redox activity of the anthraquinone units. While COFs typically exhibit moderate conductivity, their structure-property tunability and compositional diversity make them valuable candidates for hybrid capacitive systems. Conductive Metal-Organic Frameworks (MOFs), particularly 2D examples like Ni_3_(HITP)_2_, are gaining significant attention for pseudocapacitive applications. Ni_3_(HITP)_2_, noted for its high porosity and active redox centers (Xu et al., 2017), demonstrated a capacitance of 111 F g^−1^ and exceptional cycling stability over 10,000 cycles in aqueous electrolytes (Sheberla et al., 2017a). The electrochemical performance stems from the conjugated ligand system and uniform dispersion of redox-active metal sites, enabling efficient charge storage and ion accessibility. A comparative overview is presented in Table 1, summarizing recent developments in materials exhibiting redox pseudocapacitance.

**TABLE 1:** Comparison of advanced redox-active materials for pseudocapacitors.

Material	Capacitance (F/g)	Energy Density (Wh/kg)	Electrolyte	Notable Features	Ref
Ti_3_C_2_T_x_ Mxene	Up to 1500 (volumetric)	∼25–35	H_2_SO_4_	Layered structure, high conductivity	Sheberal et al. (2017a)
DAAQ-TFP COF	∼350	∼20	KOH	Redox-active quinone sites, stable cycling	Jiang et al. (2020)
Ni_3_(HITP)_2_ MOF	111	∼12	Na_2_SO_4_	Conductive π-conjugated system, 10,000 cycles	Xu et al. (2017)
MnO_2_/Graphene composite	∼1100	∼30	Na_2_SO_4_	High surface area, synergistic conductivity	Xu et al. (2017)
Fe_3_O_4_/Carbon composite	∼400	∼32	KOH	High energy density, structural stability	Niu et al. (2019)

In conclusion, redox pseudocapacitance offers a versatile and efficient mechanism for energy storage in supercapacitors. Through the integration of advanced materials such as Mxenes, COFs, MOFs, and metal oxides, researchers are able to engineer electrode systems that balance high energy and power densities with long-term stability. Continued innovations in molecular design, structural optimization, and hybridization are expected to further advance.

### 3.3 Surface adsorption (electrosorption): mechanism and material platforms

Surface adsorption, also known as electrosorption, is a charge storage process where ions from the electrolyte are adsorbed onto the surface of the electrode material without undergoing any redox reaction or penetrating the bulk of the material. While typically classified as non-Faradaic, some materials demonstrate pseudocapacitive behavior when adsorption involves partial charge transfer or specific chemical interactions (Mao et al., 2012). In this context, electrosorption represents a hybrid of electric double-layer capacitance (EDLC) and surface-confined pseudocapacitive processes, contributing significantly to the total capacitance. The overall mechanism can be represented as: M + xC + xe−↔M-Cx,​ where M is the electrode surface and C^+^ is the adsorbing cation. This process is typically reversible and diffusion-independent, enabling ultrafast charge/discharge kinetics. The capacity of such systems largely depends on surface area, porosity, and the nature of surface functional groups.

Carbon-based materials such as activated carbon, graphene, and carbon nanotubes are well-known examples of charge storage dominated by surface adsorption. However, surface functionalization can introduce pseudo-Faradaic sites that enhance electrosorption. For example, oxygen-containing groups (–OH, –COOH) on graphene oxide can interact with cations via Lewis’s acid-base or hydrogen bonding, slightly shifting the mechanism from pure EDLC to pseudocapacitive electrosorption (Hajalilou et al., 2018). In these systems, capacitance values typically range from 100 to 300 F/g, with high power densities and excellent cycling stability over 10,000 cycles.

More recently, materials like Mxenes and metal–organic frameworks (MOFs) have demonstrated surface-confined electrosorption mechanisms (Augustyn et al., 2013; Lukatskaya et al., 2013) For example, Ti_3_C_2_T_x_ Mxene films show combined EDLC and electrosorptive behavior due to the presence of surface terminations like OH and F. These groups provide localized binding sites for cation adsorption, especially in acidic and neutral electrolytes. Similarly, certain MOFs such as Cu_3_(HHTP)_2_ display large surface areas and tunable pore environments, allowing for controlled electrosorption of ions with high surface capacitance (∼110 F g^−1^) and good rate performance. The process can be described as surface ion pairing, often reversible and accompanied by a partial change in electron density distribution on the surface atoms.

Electrosorption-based charge storage has recently expanded to include functionalized 2D materials and porous organic frameworks (POFs), where the critical factors are pore size, ion solvation, and surface chemistry. Specifically, nitrogen-doped carbons from biomass demonstrated high electrosorptive capacity due to improved electronic conductivity and more active sites for ionic interactions. In KOH electrolytes, a specific capacitance of 320 F g^−1^ was achieved, showcasing the synergy between surface adsorption and tailored surface chemistry. These results emphasize the crucial role of designing materials with high surface-to-volume ratios and optimized surface functionalities for improved electrosorption.

## 4 Ni-driven pseudocapacitive batteries

Pseudocapacitive batteries represent an approach to battery design that utilizes the fast, surface-based redox reactions of materials to enhance power density and charging rates, aiming to bridge the performance gap between supercapacitors and batteries. Ni-based metal oxides have shown exceptionally high pseudocapacitive properties, ranking among the most promising transition metal oxides for energy storage applications. Ni-based electrodes have garnered significant attention due to several favorable properties, including high theoretical capacitance, environmental compatibility, chemical stability, and abundant electroactive sites. From a practical standpoint, the suitability of nickel in large-scale energy storage systems is not solely determined by electrochemical performance it must also consider factors such as material abundance, cost, and supply chain resilience. Nickel is relatively abundant in the Earth’s crust compared to critical elements like cobalt or ruthenium and is already widely used in stainless steel, coinage, and battery industries. However, the recent surge in global demand driven by electric vehicle (EV) battery production and renewable energy systems has caused fluctuations in nickel prices and raised concerns about long-term availability and sustainability. Despite this, low-to-medium-grade nickel resources are widespread and economically viable, and ongoing efforts in nickel recycling and recovery technologies are helping to stabilize supply and reduce environmental impact.

**FIGURE 4:**
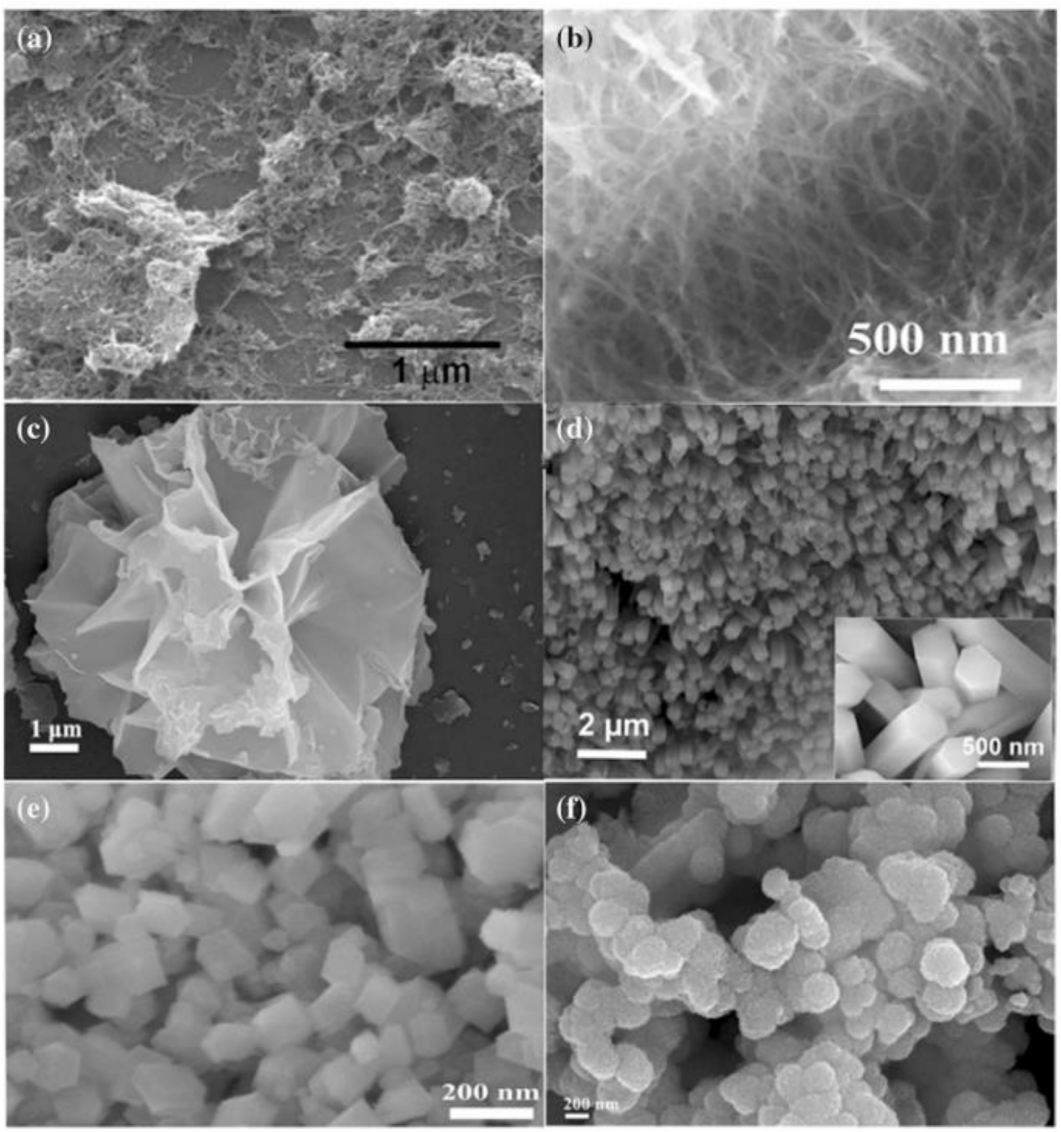
Shows an **(a,b)** SEM images of NiO–CNT and Ni@NiO nanowires. **(c,d)** SEM images of NiO@CeO_2_ and NiO micro rods. **(e,f)** SEM images of NiO and NiO/MnO_2_. Reprinted with permission from Lee et al. (2025).

Therefore, nickel remains a cost-effective and scalable option, especially for supercapacitor and pseudocapacitive battery applications where its loading per device is relatively low compared to high-energy lithium-ion batteries. Moreover, Ni-based materials, particularly in nanostructured or composite forms, offer an ideal balance of cost-performance, positioning them as competitive candidates for future energy storage technologies especially in grid-level systems and portable electronics. As a result, achieving high electrochemical values in the device requires a large electrode surface area. Various metal oxide electrode materials are deposited with nanostructured compounds to increase the electrode’s surface area, as shown in Figure 4. Furthermore, Ni-based compounds are becoming more popular because of their outstanding electrical performance and unique advantages over oxide/hydroxide-based materials. NiO-based core-shell structures have generally been successfully used in supercapacitors, showing major improvements in terms of increasing their specific capacity and cyclic stability. To achieve high pseudocapacitive performance in Ni-based materials, a primary goal is to synthesize nanostructured materials with a high specific surface area, enabling fast ion diffusion, efficient charge transfer, and high rate capability.

Furthermore, the vast majority of NiO particles generated in experiments show an ability to form. Investigators have used a number of methods to improve NiO’s electrochemical performance in reaction to the aforementioned problem. In materials synthesized process have various groups, Ni-based scanning electron microscopy exhibit unique surface morphology. The development of NiO nanosheets contributes to a more uniform distribution within the sample, effectively increasing the available surface area for electrochemical reactions. Among various transition metal oxides, NiCo_2_O_4_ stands out over NiO and Co_3_O_4_ due to its broader range of redox-active valence states and significantly higher electrical conductivity, making it a highly promising candidate for advanced supercapacitor electrodes (Kim et al., 2015). Structurally, NiCo_2_O_4_ exhibits a standard spinel configuration, where divalent nickel ions occupy tetrahedral positions, while cobalt ions existing in both +2 and +3 oxidation states are distributed across both tetrahedral and octahedral sites. This mixed-valence arrangement enhances electron transport efficiency, enabling single-crystal NiCo_2_O_4_ nanoplates to achieve electrical conductivities as high as 62 S/cm at room temperature (300 K), attributed to the low activation energy for charge movement facilitated by the coexistence of multiple oxidation states. At a current density of 0.05 A g^−1^, it provided a specific capacitance of 1125 F/g (Zhang et al., 2021; Zhu et al., 2011). Ni-based metal oxides achieving high surface area, due to their inherent advantages of electrochemical properties. Due to low cost, eco-friendliness and high theoretical capacitance further performance their capability in energy storage systems.

### 4.1 Nickel-Zinc (Ni-Zn) batteries

Ni-Zn batteries have seen a comeback in demand in recent decades because of an increasing demand for more environmentally friendly and more efficient technologies for storing energy. This study explores the synthesis and application of a novel bimetallic Ni,Zn-based metal-organic framework (MOF) and its derived hydroxide for use as battery-type electrodes in supercapacitors. The authors employed a simple, one-step electrochemical deposition technique to grow Ni,Zn-MOF thin films directly on nickel foam (NF). This method stands out for being more accessible and controllable compared to conventional solvothermal or hydrothermal routes. After deposition, the Ni,Zn-MOF was chemically converted into a hydroxide-carbon composite (Ni_1.5_Zn_0.5_(OH)_2_@C) by treating it with KOH solution. The structures of both the MOF and hydroxide derivatives were confirmed using various analytical techniques including XRD, FT-IR, FE-SEM, AFM, and EDX. Both materials exhibited flower-like 3D morphologies with porous nanosheet architectures that enhanced ion transport and electron conductivity.

Electrochemical characterization revealed that both electrodes displayed battery-type behavior, dominated by Faradaic redox reactions. In a three-electrode configuration, the Ni,Zn-MOF delivered a specific capacity of 442.9 mAh/g at 1 A/g, with 94.2% retention after 6000 cycles at 5 A/g. The hydroxide derivative exhibited even better performance, reaching 637 mAh/g and maintaining 91.9% capacity after 6000 cycles. To demonstrate practical application, the authors constructed two asymmetric supercapacitor (ASC) devices using N-doped porous graphene as the negative electrode. The devices based on Ni,Zn-hydroxide@C achieved an energy density of 34.5 Wh/kg at a power density of 624 W/kg, and retained over 92% of their capacity after 3000 cycles, showcasing excellent durability and energy performance.

An effective and scalable method for preparing high-performance MOF-based and MOF-derived electrodes, emphasizing the synergistic role of Ni and Zn in enhancing capacitive behavior. The findings highlight the strong potential of these materials for next-generation energy storage systems, particularly in asymmetric supercapacitor configurations (Li C. et al., 2017; Sheberla et al., 2017b).

### 4.2 Nickel-metal hydride (Ni-MH) batteries

A detailed evaluation of various nickel-based materials is depicted in Table 2, particularly focusing on different forms of nickel hydroxide (Ni(OH)_2_) and their oxidized derivatives (NiOOH), as well as Ni-based composites.

**TABLE 2:** Evaluation of various nickel-based materials as electrodes.

Material	Structure/Form	Redox Couple	Key Features	Capacitance/Performance	Ref.
α-Ni(OH)_2_	Rhombohedral, layered	α-Ni(OH)_2_/γ-NiOOH	Large interlayer spacing (∼0.8 nm), includes water and anions, higher theoretical capacity	Higher capacity due to 2-electron transfer	Lee et al. (2001)
β-Ni(OH)_2_	Trigonal, layered	β-Ni(OH)_2_/β-NiOOH	More stable phase, commonly used in commercial Ni-MH batteries	Lower capacity, better structural stability	Tkalych et al. (2015)
β-NiOOH	Oxidized form of β-Ni(OH)_2_	—	Hexagonal structure, interlayer spacing expands (c = 4.85 Å)	Oxidation state +3 to +3.2	Fu et al. (2007)
γ-NiOOH	Oxidized form of α-Ni(OH)_2_	—	Contains both Ni^3+^ and Ni^4+^, higher oxidation state range (3–3.7)	Higher energy storage potential	Singh and Schechter, 2018 (2018)
NiO (on carbon nanotubes)	Nanotubes	Ni^2+^/Ni^3+^ redox	Grown on multi-walled CNTs using sacrificial template method	∼245.3 F g^-1^ at unspecified current density	Zhang et al. (2025)
Co/Mn-substituted Ni(OH)_2_	Grown on rGO sheets	Ni/Co or Ni/Mn mixed redox	One-pot chemical bath synthesis, improved conductivity and redox activity	665 C g^-1^ (2 A g^-1^), 427 C g^-1^ (20 A g^-1^)	Khan et al. (2023)
Ni-Co binary oxide	Amorphous porous structure	Ni/Co redox	High surface area, improved ion transport, multiple oxidation states	1607 F g^-1^ @ 0.5 A g^-1^; 91% retention after 2000 cycles	Zhou et al. (2020b)

Among these, the two main polymorphs of nickel hydroxide, α-Ni(OH)_2_ and β-Ni(OH)_2_, exhibit distinctly different structural and electrochemical behaviors. The α-phase has a layered rhombohedral structure with large interlayer spacing (∼0.8 nm), which accommodates water and anions. This results in higher theoretical capacity due to a two-electron transfer process leading to the formation of γ-NiOOH. However, it tends to be less stable in alkaline environments Hashmi et al. (2020). In contrast, β-Ni(OH)_2_ has a more compact trigonal structure and is typically used in commercial Ni-MH batteries due to its greater structural stability, although its capacity is generally lower because it undergoes a single-electron redox process.

The oxidized forms, β-NiOOH and γ-NiOOH, represent the electrochemically active species during charge–discharge cycles. β-NiOOH results from the oxidation of β-Ni(OH)_2_ and exhibits a relatively stable structure with oxidation states of +3 to +3.2. γ-NiOOH, derived from α-Ni(OH)_2_, supports a higher oxidation state up to +3.7, contributing to its enhanced energy storage potential but at the cost of long-term stability.

In addition to pure hydroxides, the paper discusses nanostructured and composite materials like NiO grown on carbon nanotubes (CNTs) and Co/Mn-substituted Ni(OH)_2_ on reduced graphene oxide (rGO). These composites are engineered to improve conductivity, surface area, and ion transport. For instance, NiO on CNTs demonstrated a respectable capacitance of ∼245 F g^−1^, while Co/Mn-Ni(OH)_2_ composites reached up to 665 C/g at 2 A/g, showcasing both high capacity and excellent rate capability. Similarly, Ni-Co binary oxides with porous amorphous structures delivered 1607 Fg^−1^ at 0.5 Ag^−1^ and retained over 90% of their capacity after 2000 cycles, indicating their promise for long-cycle-life supercapacitors (Khan et al., 2023; Zhou et al., 2020b).

### 4.3 Nickel-iron (Ni-Fe) batteries

The development of high-performance nickel-ron (Ni-Fe) nanostructured composites anchored on a three-dimensional nitrogen-doped graphene (3DNG) support for supercapacitor applications. A series of Ni:Fe ratios (1:0, 1:1, 1:2, 1:3, 1:4, and 0:1) were synthesized and evaluated to optimize the electrochemical performance. Among these, the composite with a Ni:Fe ratio of 1:2 (P-3DNG/NiFe1:2) emerged as the optimal formulation, delivering a high specific capacitance of 300 F g^−1^ at 0.5 A g^−1^, which remained relatively stable at higher current densities with 62% retention at 8 A g^−1^. This performance is attributed to the synergistic redox interactions between Ni^2+^/Ni^3+^ and Fe^2+^/Fe^3+^, along with the conductive, high-surface-area 3DNG structure that improves electron and ion transport while preventing active material agglomeration.

To developed an innovative three-dimensional nitrogen-doped graphene/iron-nickel (3DNG/FeNi) composite, engineered as a high-performance electrode material for supercapacitor applications. The material was synthesized via a simple sol–gel approach followed by pyrolysis, yielding a structurally robust hybrid with excellent electrochemical properties. A key feature of their design is the use of azodicarbonamide as a dual-function precursor, acting both as a nitrogen dopant and as a porosity enhancer. This enabled the formation of a highly porous and conductive 3D nitrogen-doped graphene (3DNG) scaffold that provides an ideal matrix for the uniform integration of Fe–Ni bimetallic nanoparticles. Nitrogen doping not only improves the electrical conductivity but also introduces electrochemically active sites and enhances the chemical interaction between the carbon framework and metal particles. The resulting synergy between the graphene matrix and Fe-Ni nanoparticles lead to improved charge storage performance, combining electric double-layer capacitance with faradaic (pseudocapacitive) contributions.

**FIGURE 5:**
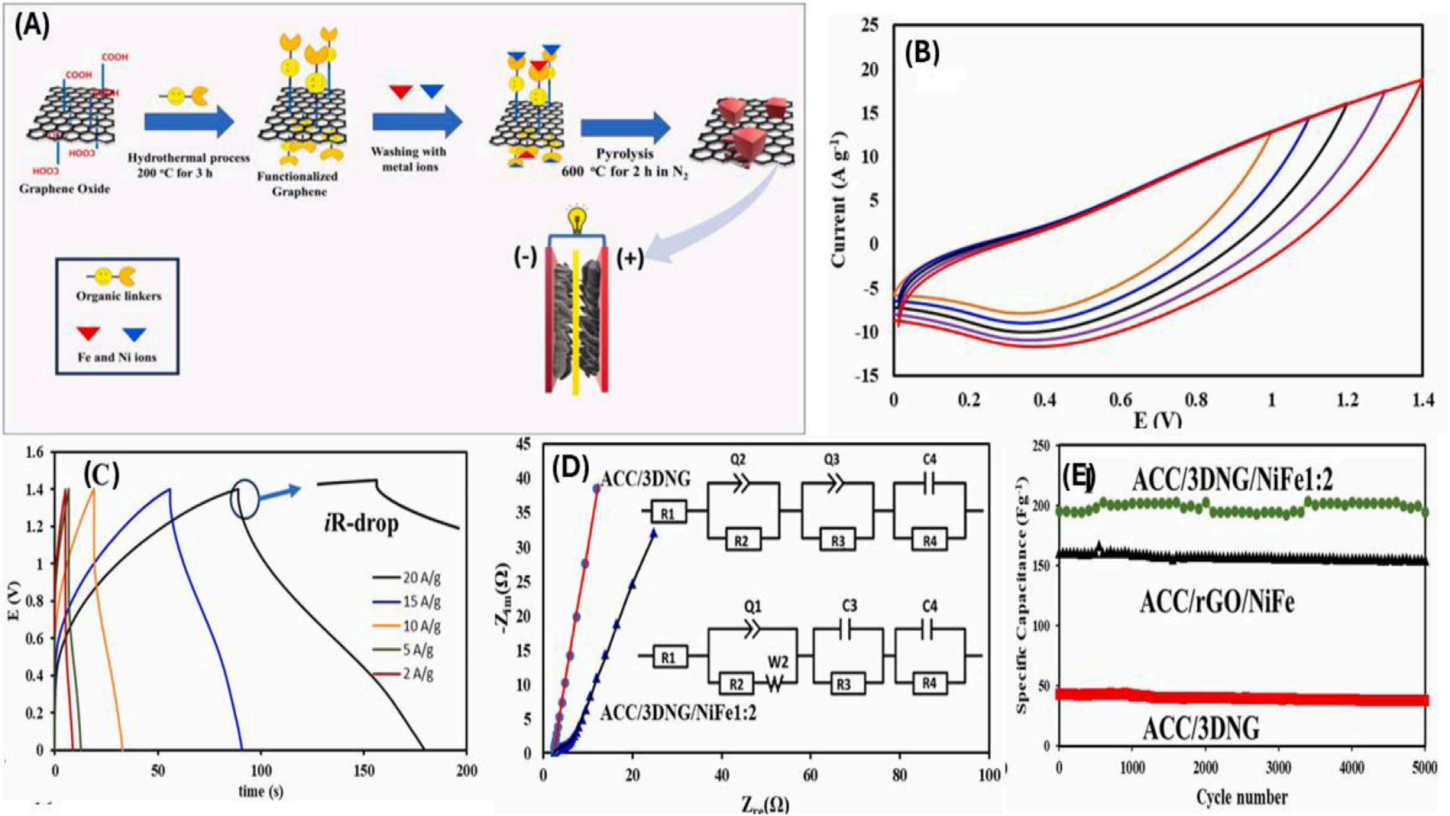
**(A)** Schematic illustration of the synthesis route for the 3D nitrogen-doped graphene/FeNi (3DNG/FeNi) composite. **(B,C)** ACC/3DNG/NiFe1:2//VC/ACC device at different window potentials, Galvanostatic charge–discharge (GCD) curves of the asymmetric supercapacitor device (ACC/3DNG/NiFe1:2//VC/ACC) at various current densities, **(D)** Nyquist plots (electrochemical impedance spectroscopy) of ACC/3DNG and ACC/3DNG/NiFe1:2 electrodes measured in the frequency range of 100 kHz to 0.01 Hz, **(E)** Cycling stability of ACC/3DNG/NiFe1:2, ACC/3DNG, and ACC/rGO/NiFe electrodes at a constant current density of 4 A g^−1^ over 5000 charge-discharge cycles. Printed with permission from Lin et al. (2013).

Electrochemical tests revealed that the 3DNG/FeNi composite exhibits high specific capacitance, excellent rate capability, and outstanding long-term cycling stability. The enhanced performance is attributed to the hierarchical porosity, which facilitates rapid ion transport and effective electrolyte diffusion, and the homogeneous dispersion of Fe-Ni nanoparticles, which promotes stable redox activity. To demonstrate its practical applicability, the authors assembled an asymmetric supercapacitor (ASC) device using the 3DNG/FeNi (with a Ni:Fe ratio of 1:2) as the positive electrode and commercial Vulcan carbon (VC) as the negative electrode. This device achieved a high energy density of 35.4 Wh kg^−1^ and an impressive power density of 14 kW kg^−1^, outperforming many conventional carbon-based systems as shown in Figure 5. Furthermore, the device maintained excellent cycling performance at a current density of 4 Ag^−1^, confirming its potential for long-term energy storage applications.

Electrochemical impedance spectroscopy (EIS) revealed reduced charge-transfer resistance in the FeNi-integrated electrode, further validating the role of metal nanoparticles in facilitating electron transport. Nyquist plots and equivalent circuit modeling supported these findings, while galvanostatic charge–discharge (GCD) measurements at various current densities confirmed the device’s rapid power response and stable performance across a wide voltage window. Overall, this study highlights the advantages of integrating nitrogen-doped carbon nanostructures with redox-active transition metal alloys. The 3DNG/FeNi composite presents a scalable and efficient route toward the fabrication of advanced electrode materials, with potential applications in high-power energy storage systems such as electric vehicles, grid support modules, and portable electronics.

The material demonstrated excellent long-term stability, maintaining 97% of its original capacitance even after 5,000 charge-discharge cycles, highlighting its robust electrochemical resilience. When assembled into an asymmetric supercapacitor using Vulcan carbon (VC) as the negative electrode, the NiFe1:2 composite delivered an energy density of 35.4 Wh/kg and a power output of 14 kW/kg. Additionally, the device retained 86% of its capacitance after 10,000 cycles. These findings point to the material’s strong potential for use in high-performance, rapid-charging energy storage systems. Composites with other Ni:Fe ratios, such as 1:3 or 1:1, showed moderate performance, while excessive iron content (1:4) or the use of Fe-only electrodes led to performance degradation. The data emphasize that a precise balance of Ni and Fe is critical for achieving optimal electrochemical activity. The study highlights the promise of Ni-Fe-graphene hybrids as scalable, stable, and high-performing electrode materials for next-generation supercapacitors (Tahalyani et al., 2020; Amir et al., 2025).

### 4.4 Nickel-cadmium (Ni-Cd) materials

Traditional Ni-Cd batteries, although less favored today due to the environmental concerns surrounding cadmium, remain of academic interest due to their robust redox chemistry and cycle stability. In this context, nickel-based materials such as NiO nanowires and nanofibers are highlighted for their high surface area and efficient ion diffusion pathways. Specifically, 1D NiO nanowires facilitate fast electron transport, while electrospun NiO nanofibers on nickel foam substrates offer a porous, binder-free structure that enhances contact with the current collector and supports rapid redox kinetics.

In efforts to boost performance further, composite materials such as NiO@CdS core-shell structures have been developed. These heterostructures combine the redox activity of NiO with the high conductivity and photoactivity of CdS, resulting in improved charge storage behavior compared to the individual components alone is depicted in Table 3. Although specific capacitance values for these Ni-Cd composites were not always quantified in the paper, the structural advantages suggest strong potential for enhanced performance.

**TABLE 3:** Evaluation of various NiO-based materials used in Ni-Cd batteries.

Materials	Structure	Specific Capacitance	Electrochemical Performances
NiO Nanowires	1D nanostructures	Not specified in this table	Excellent for fast electron transport and efficient ion diffusion
NiO Nanofibers (Electrospun)	Porous interconnected network on Ni foam	Not specified in this table	Binder-free electrodes; high contact with current collector
Ni(OH)_2_	Various nanostructures (e.g., nanosheets, nanoflakes)	Up to ∼2000+ F/g^-1^ (in other sources)	Key redox reactions: Ni(OH)_2_ + OH^−^ ↔ NiOOH + H_2_O+ e^−^; affected by doping and structure
NiO@CdS Core–Shell	Core-shell heterostructures	Enhanced compared to bare NiO or CdS	CdS improves conductivity and redox activity when used with NiO

Furthermore, Ni(OH)_2_, a key component in Ni-Cd electrochemistry, is referred as a material with tunable morphology (e.g., nanosheets, nanoflakes) and a theoretical specific capacitance exceeding 2000 F/g^-1^ in optimal conditions. Its well-known redox couple (Ni^2+^/Ni^3+^) makes it a reliable and effective pseudocapacitive material, especially when nanostructured or doped to improve conductivity and cycle life (Bastakoti et al., 2013).

## 5 Conclusion and outlook

Pseudocapacitive materials represent a promising class of advanced electrode materials for supercapacitors (SCs), utilizing mechanisms such as ion intercalation, surface redox reactions, and adsorption-based charge storage. Over the past decade, significant progress has been made in engineering high-performance pseudocapacitive electrodes, despite persistent challenges like material degradation, poor conductivity, and active layer detachment during prolonged cycling. Nickel-based compounds, with their multiple oxidation states and high theoretical capacitance, have emerged as cost-effective and high-performing candidates. Likewise, newer materials like Mxenes and MOFs have enhanced interfacial contact and charge transfer through structural engineering. Traditional materials such as transition metal oxides (e.g., RuO_2_, MnO_2_) and conductive polymers (e.g., polyaniline, polypyrrole) continue to serve as key platforms for understanding and improving pseudocapacitive behavior. Looking ahead, pseudocapacitive materials are expected to play pivotal roles in a range of practical energy storage scenarios, including: Fast-charging power tools and electric vehicles, where high-rate capability is essential, Grid-level energy buffering for renewable energy integration, Wearable and flexible electronics, which require lightweight, high-capacity storage, Low-temperature or harsh-environment electronics, due to the robust charge/discharge behavior of pseudocapacitors.

(1) Material Synthesis and Fabrication. Future advancements will hinge on designing hierarchical nanostructures and binder-free electrodes that optimize electroactive surface area, conductivity, and mechanical stability. Tailoring the chemical composition and surface functionality of pseudocapacitive materials will be crucial for improving their scalability and real-world applicability.
(2) Advanced Characterization Technologies. In-depth understanding of pseudocapacitive mechanisms requires real-time and atomic-level insights into structural changes during operation. Advanced techniques such as *in-situ* XRD, TEM, STEM, and XAS are enabling researchers to precisely correlate material design with electrochemical behavior.
(3) Performance Optimization Through Computation. Computational modeling including first-principles DFT and molecular dynamics simulations offers predictive capabilities to guide synthesis routes, identify favorable ion insertion sites, and evaluate thermodynamic stability, accelerating the discovery of next-generation electrode materials.
(4) The AI Revolution in Energy Storage. Artificial Intelligence (AI) is rapidly transforming the discovery and optimization of pseudocapacitor systems. From high-throughput material screening to predictive modeling of performance and failure mechanisms, AI enhances efficiency, reduces costs, and shortens development timelines. Furthermore, AI-guided process control in synthesis and manufacturing can improve consistency and scalability.

In conclusion, continued interdisciplinary research combining materials science, electrochemistry, computational modeling, and AI will be key to unlocking the full potential of pseudocapacitive materials. Their unique balance of power and energy performance makes them strong contenders in a future dominated by rapid, efficient, and sustainable energy storage solutions.
